# Z-ligustilide restores tamoxifen sensitivity of ERα negative breast cancer cells by reversing MTA1/IFI16/HDACs complex mediated epigenetic repression of ERα

**DOI:** 10.18632/oncotarget.16440

**Published:** 2017-03-22

**Authors:** Hui Ma, Li Li, Guojun Dou, Chengqiang Wang, Juan Li, Hui He, Mingxia Wu, Hongyi Qi

**Affiliations:** ^1^ College of Pharmaceutical Sciences, Southwest University, Chongqing 400716, China

**Keywords:** Z-ligustilide, tamoxifen, ERα negative breast cancer, MTA1, histone modification

## Abstract

Emerging evidence indicates epigenetic modification represses estrogen receptor α (ERα) and contributes to the resistance to tamoxifen in aggressive ERα-negative (ERα^−^) breast cancer. Z-ligustilide is a major compound in *Radix Angelica sinensis*, an herb from traditional Chinese medicine (TCM) most frequently prescribed for breast cancer. However, the role of Z-ligustilide in ERα^−^ breast cancer and epigenetic modification remains largely unknown. Herein we showed, for the first time, that Z-ligustilide restored the growth inhibition of tamoxifen on ERα^−^ breast cancer cells. Apoptosis and S and G2/M phases cell cycle arrest were induced by combinatorial Z-ligustilide and tamoxifen. Importantly, Z-ligustilide reactivated the ERα expression and transcriptional activity, which is proved to be indispensable for restoring the sensitivity to tamoxifen. Interestingly, Z-ligustilide increased Ace-H3 (lys9/14) enrichment in the ERα promoter. Moreover, Z-ligustilide dramatically reduced the enrichment of metastasis-associated protein 1 (MTA1) as well as IFN-γ-inducible protein 16 (IFI16) and histone deacetylases (HDACs) onto the ERα promoter. Meanwhile, Z-ligustilide downregulated MTA1, IFI16 and HDACs, which caused destabilization of the corepressor complex. Collectively, our study not only highlights Z-ligustilide as a novel epigenetic modulator, but also opens new possibilities from TCM for treating aggressive tamoxifen-resistant breast cancer.

## INTRODUCTION

Breast cancer is one of the most common malignant tumors in women. It's estimated that the new diagnoses of breast cancer in women of United States will be 246,660 cases in 2016 and rank the first place in all new cancer diagnoses in women. The breast cancer deaths in women are expected to 40, 450 in 2016, which is next only to cancer of the lung and bronchus [1]. In China, breast cancer is also the leading cause of female malignant tumors in the statistical years of 2000 to 2011 with an increasing trend. Moreover, mortality from breast cancer rose progressively during the past three decades [2, 3]. Out of the total breast cancers, approximately 30 to 40 % of women with breast cancer belong to estrogen receptor alpha negative (ERα^−^) breast cancer, which is characterized by more aggressive phenotype, poor prognosis and recalcitrance to conventional hormonal therapies [4].

Tamoxifen (TAM), the most common hormonal therapy, is effective for both early and advanced estrogen receptor alpha positive (ERα^+^) breast cancer in pre- and post-menopausal women [5]. The anti-tumor effect of TAM is well-established due to its antiestrogenic activity. In ERα^+^ breast cancer cells, estrogen binds to ERα forming a complex. Subsequently, the complex homodimerizes and binds to the estrogen response elements (ERE) of estrogen-sensitive genes, which renders unlimited and uncontrolled cell proliferation. TAM competitively inhibits the binding of estrogen to ERα. As a consequence, the expression of estrogen-sensitive genes was inhibited by TAM, which finally results in the cell cycle arrest and a slowing of cell proliferation [6, 7]. On the contrary, ERα^−^ breast cancers are resistant to TAM. It's well documented that the etiology of the absence of ERα is rarely due to mutations, deletions, loss of heterozygosity, or polymorphisms within the gene [4, 8]. Emerging evidence over the last decades suggests that hypermethylation and histone acetylation/deacetylation in the ERα promoter are implicated as a common mechanism responsible for ERα gene repression in ERα^−^ breast cancer cells [9, 10]. Supportively, several well-characterized pharmacologic inhibitors of DNA methylation such as 5-aza-20-deoxycytidine (5-aza-dc) and histone deacetylation such as trichostatin A (TSA) were demonstrated to reactivate ERα expression in ERα^−^ breast cancer cells, respectively [11–13]. Furthermore, the distinct corepressor complexes containing transacting factors have also been shown to form on the promoter regions of ERα gene [10]. For instance, the pRb2/p130-multimolecular complex [14] and Twist recruited the HDAC1 and DNMT3B repressor complex [15] were found to occupy the ERα promoter, resulting in transcriptional repression of ERα gene. Metastasis-associated protein 1 (MTA1), a component of the nucleosome remodeling and histone deacetylase (NuRD) complex, is well known as a repressor of the transactivation function of ERα [16]. Recent study discovered that MTA1 complex including the trans-acting factor IFI16 and class II HDACs was recruited to the ERpro315 region of the ERα promoter, resulting in repression of ERα expression and generation of TAM resistance in MDA-MB-231 cells, highlighting that targeting the MTA1-IFI16 repressor complex may provide an alternative way for sensitizing ERα^−^ breast cancer cells to TAM-based chemotherapies [17].

*Angelica sinensis* (Oliv.) Diels (Apiaceae) is a valuable medicinal and edible plant in traditional Chinese medicine (TCM). *Radix Angelica sinensis* is the dried root of *Angelica sinensis* and named as dang gui (Chinese) or dong quai (English) [18]. In TCM, *Radix Angelica sinensis* is usually used for gynecological disorders. Statistically, Chinese herbal products containing *Radix Angelica sinensis* are the most frequently prescribed in Taiwan for breast cancer [19]. Further population-based study indicates that almost half of TAM-treated breast cancer survivors had taken *Radix Angelica sinensis*. Notably, consumption of *Radix Angelica sinensis* decreased the risk of subsequent endometrial cancer among breast cancer survivors aged 20-79 years after TAM treatment [20]. Although pharmacological studies revealed that *Radix Angelica sinensis* exhibited dramatically inhibitory effect on different tumors over the last two decades [21–26], its effect on breast tumor, especially on TAM-based chemotherapies, is still largely unknown. Z-ligustilide (Z-LIG) is a representative compound accounting for more than 50 % in the volatile oil of *Radix Angelica sinensis* (VORAS) [27] and also responsible for the strong aromatic odor of *Radix Angelica sinensis* [28]. Emerging evidence indicates Z-LIG has the anti-tumor effect on colorectal cancer [22] and prostate cancer [29], leukemia [26] and brain tumor [23]. However, nothing is yet known of its effect on breast cancer. Moreover, it has been shown that Z-LIG is able to reactivate nuclear factor-erythroid-2-related factor 2 (Nrf2), a key regulator of cellular antioxidant defense, by the epigenetic modification mechanism in murine prostate cancer TRAMP C1 cells [29]. Thus, it's very interesting to us that whether Z-LIG could reactivate ERα expression via epigenetic modification and then restore TAM sensitivity of ERα^−^ breast cancer cells.

In the current study, we first determined the growth inhibition of combinatorial Z-LIG and TAM in three different ERα^−^ breast cancer cell lines. Whether this combination induced apoptosis and cell cycle arrest was further investigated. Subsequently, we determined the influence of Z-LIG on ERα expression and transcriptional activity. Moreover, the effect on acetylation of histone in the ERα promoter region exerted by Z-LIG was also determined. Finally, the role of MTA1/IFI16/HDACs corepressor complex in Z-LIG mediated re-expression of ERα was specially examined.

## RESULTS

### Combinatorial Z-LIG and TAM suppressed the growth of ERα^−^ breast cancer cells

**Figure 1:**
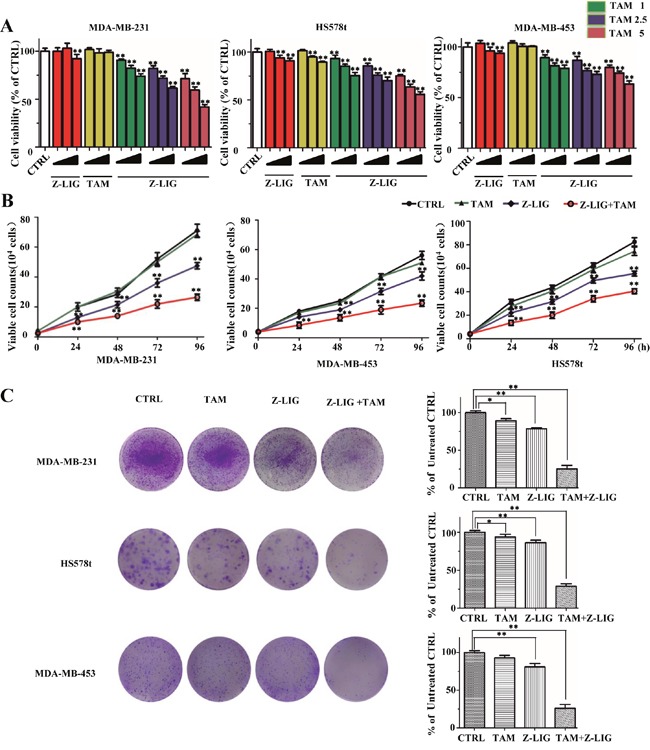
Inhibitory effect of Z-LIG and TAM alone or combination on ERα^−^ breast cancer cells **(A)** MDA-MB-231, Hs578t and MDA-MB-453 were pretreated with various concentrations of Z-LIG (10, 25, and 50 μM) for 12 h, then, cells exposed with or without TAM (1, 2.5, and 5 μM) for an extra three days and cell viability was determined by SRB assay. **(B)** Proliferation was measured by trypan blue exclusion assay. The cells growth curve represents the effect of Z-LIG (50 μM) and TAM (5 μM) alone or their combination for four days. **(C)** Colonies in three ERα^−^ breast cancer cells were treated with Z-LIG (25 μM) and TAM (2.5 μM) alone or their combination and allowed to grow for two weeks before stained with 0.005% crystal violet. Values represent mean ± SD. *p< 0.05, **p< 0.01 compared with control.

In our preliminary study, the effect of VORAS on cell viability of three different ERα^−^ breast cancer cell lines (MDA-MB-231, MDA-MB-453 and HS578t) was determined by SRB assay. As shown in Supplementary Figure 1, VORAS (20 μg/ml) and TAM (5 μM) alone exhibited no obvious cytotoxicity to all these three ERα^−^ breast cancer cells compared with CTRL (*p* > 0.05). Notably, combined treatment of VORAS with TAM induced a significant inhibitory effect on the cell viability of all these three cell lines. Moreover, MDA-MB-231 cells were more sensitive than the other two cell lines. This result indicates that VORAS can sensitize ERα^−^ breast cancer cells to TAM. Then, we asked whether Z-LIG, the main component in VORAS, has a similar effect. Supplementary Figure 2 showed that Z-LIG (10 to 400 μM) concentration-dependently inhibited the cell viability of MDA-MB-231 cells (IC_50_ = 133.6 μM). 10, 25 and 50 μM of Z-LIG were selected for the following experiments as no or only weak cytotoxicity was induced under these concentrations. The inhibitory effect of Z-LIG (10, 25 and 50 μM) and TAM (1, 2.5 and 5 μM) alone or their combination on cell viability was first determined by SRB assay in these three ERα^−^ breast cancer cell lines. As a result, Z-LIG and TAM alone showed no or only weak inhibition on all these three cell lines compared with CTRL (Figure 1A). However, combination of Z-LIG and TAM remarkably inhibited the cell viability of all these three cell lines in a concentration-dependent manner (*p* < 0.01). Similarly, MDA-MB-231cells was more sensitive to Z-LIG than the other two cell lines. Then, we further characterized the inhibitory effect of the combination of Z-LIG and TAM by determining their influence on the proliferation and the colony formation. As shown in Figure 1B, TAM (5 μM) alone showed no or only very weak inhibitory effect on the proliferation of all these three cell lines compared with CTRL, whereas Z-LIG (50 μM) alone showed moderate inhibitory effect. Expectedly, Z-LIG combined with TAM inhibited the proliferation of all these three cell lines (*p* < 0.01). Further colony formation assay also showed that Z-LIG combined with TAM remarkably reduced both the colony number (*p* < 0.01) (Figure 1C). These results suggest that Z-LIG effectively restored the sensitivity of ERα^−^ breast cancer cells to TAM.

### Combinatorial Z-LIG and TAM induced cell apoptosis in ERα^−^ breast cancer cells

**Figure 2:**
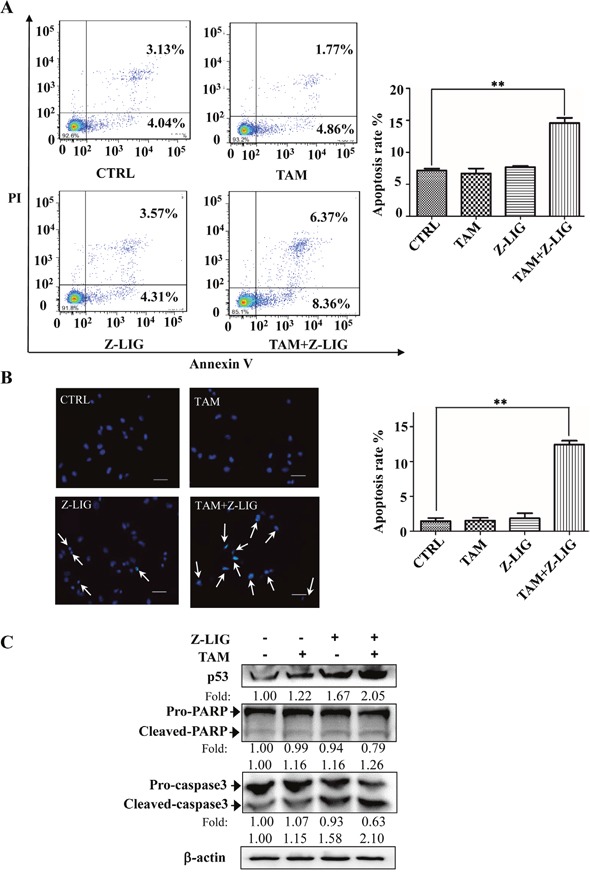
Combinatorial Z-LIG and TAM induced cell apoptosis MDA-MB-231 cells were treated with Z-LIG (50 μM) and TAM (5 μM) alone or their combination for 72 h. **(A)** Apoptotic cells were quantified by flow cytometry after stained with FITC-conjugated Annexin V and PI. **(B)** Morphologic change of apoptotic cells was evaluated by Hoechst 33342 staining. The scar bar is 50 μm. **(C)** The expression of apoptosis-related proteins as indicated were determined by Western blotting. β-actin was used as an internal control. The blots were a representative of three independent experiments. Values represent mean ± SD. *p< 0.05, **p< 0.01 compared with control.

To elucidate the molecular mechanisms underneath combinatorial Z-LIG and TAM-mediated cell growth inhibition, cell apoptosis with Annexin V/PI staining analysis was evaluated using flow cytometric analysis. As shown in Figure 2A, TAM (5 μM) and Z-LIG (50 μM) alone showed no stronger inducing effect on apoptosis compared with CTRL (*p*>0.05). Notably, combinatorial Z-LIG and TAM induced almost two times increase of apoptotic rate compared with that of control (*p* < 0.01). Furthermore, we used Hoechst 33342 staining to detect the morphologic change of apoptotic cells in MDA-MB-231. As shown in Figure 2B, the combined group exhibited much more cells with condensed and fragmented nuclei than control (*p* < 0.01). To further characterize the apoptosis-inducing effect, we determined the apoptosis-related proteins in MDA-MB-231 cells after treated by Z-LIG (50 μM) and TAM (5 μM) alone or their combination with western blotting analysis. Figure 2C demonstrated that Z-LIG combined with TAM remarkably induced p53 expression and promoted the conversion of pro-PARP and pro-caspase 3 to cleaved-PARP and cleaved-caspase 3, respectively, whereas Z-LIG and TAM alone exhibited only relative weaker effects. In Supplementary Figure 3, we further confirm the involvement of p53 and found that combinatorial Z-LIG and TAM also markedly induced p53 expression in MDA-MB-453 and HS578t cells. These results revealed that Z-LIG combined with TAM induced apoptosis of ERα^−^ breast cancer cells and the cell growth inhibition mediated by this combination may, at least in part, be due to apoptosis.

### Combinatorial Z-LIG and TAM induced cell cycle arrest in ERα^−^ breast cancer cells

**Figure 3:**
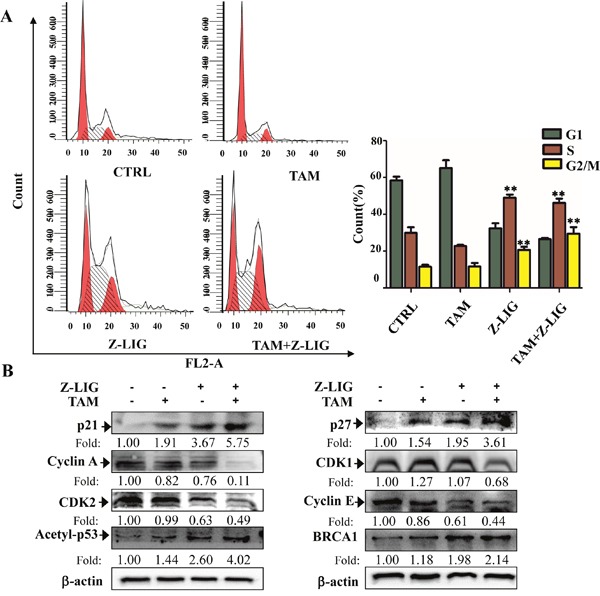
Combinatorial Z-LIG and TAM induced cell cycle arrest MDA-MB-231 cells were treated with Z-LIG (50 μM) and TAM (5 μM) alone or their combination for 72 h. **(A)** Cell cycle analysis of MDA-MB-231 cells stained with PI and analyzed by flow cytometry after the indicated treatments. **(B)** The expression of cell cycle-related proteins as indicated were determined by Western blotting. β-actin was used as an internal control. The western blots were a representative of three independent experiments. Values represent mean ± SD. *p< 0.05, **p< 0.01 compared with control.

As combinatorial Z-LIG and TAM exhibited significant anti-proliferative effect, we then analyzed influence of the combination on the cell cycle distribution of MDA-MB-231 cells. As shown in Figure 3A and Supplementary Table 1, we found that the combination decreased the percentage of cells at G1 phase from 57.99 % to 26.57 %. Specifically, compared with the untreated control cells, the percentage of cells in S phase increased from 31.01 % to 44.79 % upon the combination treatment. Moreover, G2/M phase was also induced with the percentage of these cells increasing from 11.00 % to 28.63 %. Of note, Z-LIG also induced the percentage of cells in S phase increasing from 31.01 % to 50.51% and G2/M phase increasing from 11.00 % to 19.41 %. This is in line with the moderate anti-proliferative effect of Z-LIG shown in Figure 1B.

To determine the mechanisms by which combinatorial Z-LIG and TAM triggers the S and G2/M phase arrest, we measured the expression level of cell cycle signaling proteins with Western blotting analysis. Cyclin dependent kinase (CDK) and cyclins complexes are of great significance in regulating cell cycle progression. Reciprocally, p21 and p27, CDK inhibitors, are negative regulators of cell cycle progression [30]. Figure 3B showed that cyclin A, cyclin E, CDK1 and CDK2 levels obviously decreased, while p21 and p27 levels remarkably increased in MDA-MB-231 cells as compared with CTRL after treated with Z-LIG combined with TAM. On the contrary, TAM exerted only weak or no effect on these proteins, while Z-LIG exhibited moderate effect. These results are well consistent with their influence on cell cycle arrest. Furthermore, p21 is one of the transcriptional targets of acetylation of p53 (acetyl-p53) and BRCA1 [31–33]. Our results showed that acetyl-p53 and BRCA1 protein level were significantly induced in MDA-MB-231 cells treated by combinatorial Z-LIG and TAM. On the whole, we can therefore infer that Z-LIG combined with TAM results in cell cycle arrest in the S and G2/M phases.

### Z-LIG reactivated ERα expression and transcriptional activity in ERα^−^ breast cancer cells

**Figure 4:**
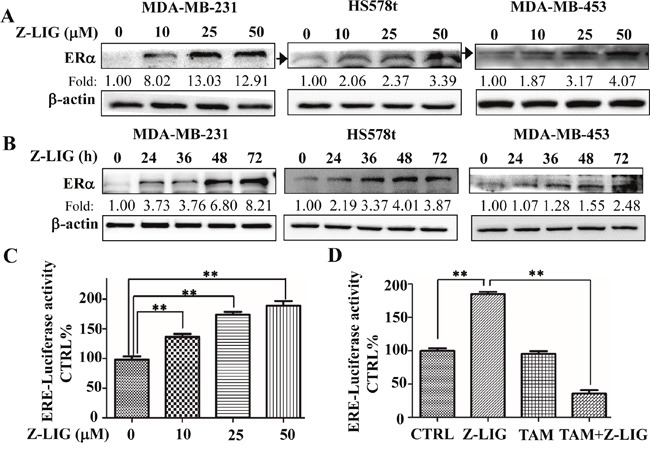
Z-LIG reactivated ERα expression and transcriptional activity in ERα^−^ breast cancer cells **(A-B)** The expression of ERα protein in MDA-MB-231, Hs578t and MDA-MB-453 cells was determined by Western blotting after treatment with Z-LIG at different concentrations and time points. The western blots were a representative of three independent experiments. β-actin was used as an internal control. **(C-D)** The transcriptional activities of ERα was examined in MDA-MB-231 cells by luciferase reporter gene assay. Cells were transfected with the ERE-luciferase plasmids construct, then treated with Z-LIG with indicated concentrations or Z-LIG (50 μM) and TAM (5 μM) alone or their combination for 72 h and finally evaluated with the Dual-Luciferase Reporter Assay System. The transcriptional activity was presented as percentage of control. Values are presented as the mean ± SD of three independent experiments. *p< 0.05, **p< 0.01 compared with control.

As the loss of ERα expression is the main reason leading to ERα^−^ breast cancer cells resistant to TAM [6, 7], we then asked whether VORAS and Z-LIG could restore the ERα expression in ERα^−^ breast cancer cells. Firstly, the three ERα^−^ breast cancer cells, MDA-MB-231, MDA-MB-453 and HS578t, were treated with various concentrations of VORAS or Z-LIG for 72 h and ERα protein expression was determined by Western blotting analysis. Expectedly, ERα protein expression was dramatically induced by VORAS in a concentration-dependent way in all these three ERα^−^ breast cancer cells (Supplementary Figure 4). Importantly, Z-LIG significantly restored the ERα protein expression in all these three ERα^−^ breast cancer cells in both concentration and time-dependent way. (Figure 4A and 4B).

In ERα^+^ breast cancer cells, ERα regulates the estrogen-sensitive genes by binding to the specific estrogen-responsive elements (ERE) and recruiting coactivators and cofactors that enhance the related downstream gene transcription [6, 7]. To further evaluate the transcriptional activity of re-expression ERα in ERα^−^ breast cancer cells, the plasmid ERE-luc was transfected into MDA-MB-231 cells. The firefly luciferase activity after MDA-MB-231 cells treated by Z-LIG and TAM alone or their combination was determined and normalized to the Renilla activity. As a result, Z-LIG activated ERE transcriptional activity in a concentration-dependent way (*p* < 0.01) (Figure 4C). Moreover, although TAM (5μM) alone showed no obvious effect on the ERE transcriptional activity, combinatorial Z-LIG and TAM significantly inhibited the ERE transcriptional activity (*p* < 0.01) (Figure 4D). These results suggest that Z-LIG significantly reactivated the transcriptional activity of ERα in ERα^−^ breast cancer cells.

### ICI182780 and si-ERα reversed the growth inhibition of combinatorial Z-LIG and TAM

**Figure 5:**
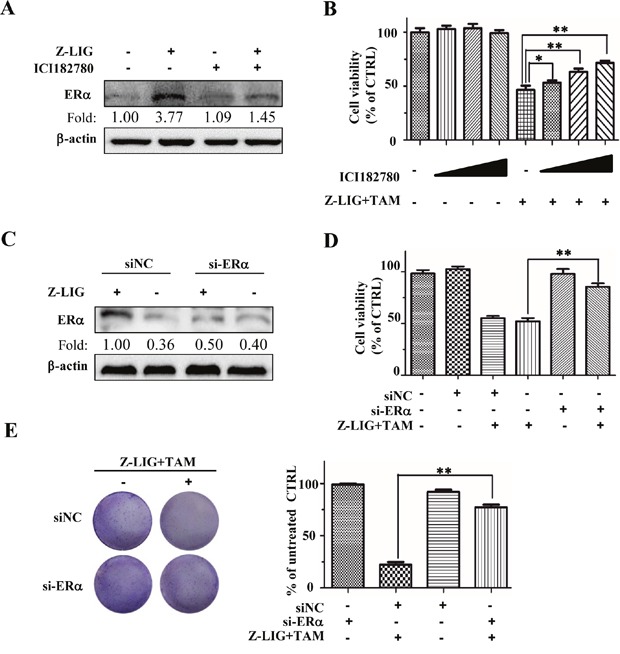
ICI and si-ERα reversed the growth inhibition of Combinatorial Z-LIG and TAM in MDA-MB-231 cells **(A)** MDA-MB-231 cells were pre-treated with 100 nM ICI182780 or vehicle for 12 h, and then with or without Z-LIG (50 μM) for another 72 h. ERα expression was analyzed by Western blotting. **(B)** The cell viability was detected by SRB assay. Cells were pre-treated with vehicle or different concentrations of ICI (10,100,1000 nM) for 12h and then treated with or without Z-LIG (50 μM) combined with TAM (5 μM) for another 72h. **(C)** The expression of ERα was measured by Western blotting in si-NC or si-ERα-transfected MDA-MB-231 cells treated with or without Z-LIG (50 μM) for 72 h. **(D)** The cell viability was detected by SRB assay after MDA-MB-231 cells were transfected with si-NC or si-ERα, and then treated with or without Z-LIG (50 μM) combined with TAM (5 μM) for another 72h. **(E)** Colony formation assay was performed in MDA-MB-231 cells that first transfected with si-NC or si-ERα, and then treated with combinatorial Z-LIG (25 μM) and TAM (2.5 μM) and allowed to grow for two weeks before stained with 0.005% crystal violet. Values represent mean ± SD. The blots or images were a representative of three independent experiments. *p< 0.05, **p< 0.01 compared with control.

To confirm whether the growth inhibition of combinatorial Z-LIG and TAM on ERα^−^ breast cancer cells is associated with the restoration of ERα protein expression, we then determined the effect of combinatorial Z-LIG and TAM on the growth of MDA-MB-231 cells after inhibition of ERα expression. Firstly, we used ICI 182780 (ICI), which is a pharmacological inhibitor of ERα and also known as Fulvestran [34]. The Western blotting result in Figure 5A demonstrated that ICI led to the significant degradation of ERα restored by Z-LIG compared with Z-LIG alone. Further cell viability assay showed that ICI concentration-dependently prevented the effect of combinatorial Z-LIG and TAM on the cell viability of MDA-MB-231 cells (Figure 5B). Then, si-ERα was applied to inhibit the ERα expression. Figure 5C showed that re-expression of ERα by Z-LIG was obviously reduced by si-ERα. Meanwhile, the MDA-MB-231 cells with si-ERα transfection exhibited more resistant to combinatorial Z-LIG and TAM compared with those only with control siRNA transfection (*p*<0.01) (Figure 5D). Furthermore, the colony formation assay also demonstrated that inhibition of ERα expression by si-ERα remarkably reversed the suppression of combinatorial Z-LIG and TAM on the colony number of MDA-MB-231 cells (*p* < 0.01) (Figure 5E). These results indicate that restoration of ERα expression by Z-LIG directly contributes to the sensitization of ERα^−^ breast cancer cells to TAM.

### Z-LIG led to histone modification changes in the ERα promoter region in ERα^−^ breast cancer cells

**Figure 6:**
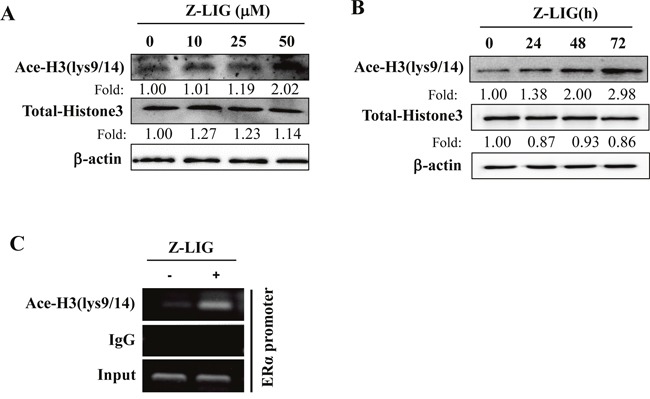
Z-LIG enhanced Ace-H3(lys9/14) expression and recruitment onto the ERα promoter in MDA-MB-231 cells **(A-B)** Expression of Ace-H3(lys9/14) and Total-Histone 3 was detected by Western blotting after MDA-MB-231 cells were treatment with Z-LIG as indicated for 72 h or Z-LIG (50 μM) for indicated times. **(C)** ChIP analysis showing recruitment of Ace-H3(lys9/14) onto ERpro315 of the ERα promoter. MDA-MB-231 cells were treated with Z-LIG (50 μM) for 72 h. DNA fragments that immunoprecipitated by normal IgG or anti-Ace-H3(lys9/14) antibodies were amplified by PCR using primers for ERpro315. The blots were a representative of three independent experiments.

Previous studies have shown that histone modification plays a crucial role in the epigenetic control of ERα expression in ERα^−^ breast cancer cells [14, 17]. To further clarify the molecular mechanisms underlying the restoration of ERα expression by Z-LIG, we first examined the effect of Z-LIG on acetylation status of histone H3 (Ace-H3), which has been demonstrated to be commonly depleted in the promoter region of ERα gene in ERα^−^ breast cancer cells [14, 17]. As shown in Figure 6A, Z-LIG, especially at 50 μM, enhanced the Ace-H3 (lys9/14) in MDA-MB-231 cells. Meanwhile, Z-LIG also time-dependently enhanced the Ace-H3 (lys9/14) level in MDA-MB-231 cells (Figure 6B). Subsequently, we determined Ace-H3 in the promoter region of the ERα gene using ChIP. Compared with control, Ace-H3 (lys9/14) was significantly enriched in the ERα promoter region in MDA-MB-231 cells treated with 50 μM of Z-LIG (Figure 6C).

### Z-LIG decreased MTA1 expression and its recruitment to the ERα promoter

**Figure 7:**
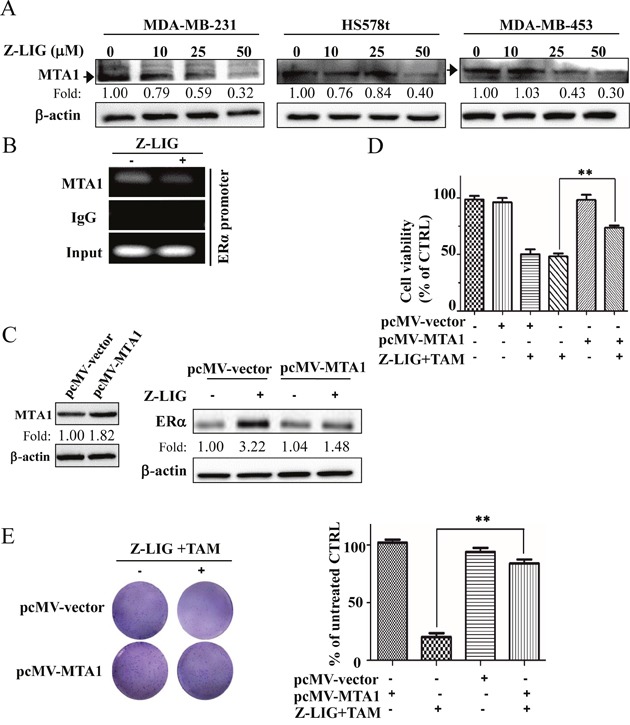
Z-LIG decreased MTA1 expression and recruitment onto the ERα promoter in ERα^−^ breast cancer cells **(A)** Expression of MTA1 was detected by Western blotting in MDA-MB-231, Hs578t and MDA-MB-453 cells after treated with Z-LIG for 72 h. **(B)** ChIP analysis showing recruitment of MTA1 onto ERpro315 of the ERα promoter. MDA-MB-231 cells were treated with Z-LIG (50 μM) for 72 h. DNA fragments that immunoprecipitated by normal IgG or anti-MTA1 antibodies were amplified by PCR using primers for ERpro315. **(C)** Expression of MTA1 was detected by Western blotting after MDA-MB-231 cells were transfected with pcMV-vector or pcMV-MTA1 for 24 h and then treated with Z-LIG (50 μM) for 72 h. **(D)** The cell viability was detected by SRB assay after MDA-MB-231 cells were transfected with pcMV-vector or pcMV-MTA1, and then treated with or without Z-LIG (50 μM) combined with TAM (5 μM) for 72 h. **(E)** Colony formation assay was performed in MDA-MB-231 cells that first transfected with pcMV-vector or pcMV-MTA1, and then treated with combinatorial Z-LIG (25 μM) and TAM (2.5 μM) and allowed to grow for two weeks before stained with 0.005% crystal violet. Values represent mean ± SD. The blots or images were a representative of three independent experiments. *p< 0.05, **p< 0.01 compared with control.

Previous study has been shown that MTA1 is negatively related to ERα expression and its recruitment to the ERpro315 region of the ESR1 promoter contributes to the epigenetic repression of ERα expression in ERα^−^ breast cancer cells [17]. To address the potential role of MTA1 in transcriptional control of Z-LIG-mediated ERα expression, we first examined the level of MTA1 expression in the ERα^−^ MDA-MB-231, MDA-MB-453, HS578t cells after treated with various concentrations of Z-LIG for 72 h. As shown in Figure 7A, there is a relatively high basal level of MTA1 in all these three ERα^−^ breast cancer cells. However, Z-LIG significantly decreased the MTA1 expression in a concentration-dependent way in all these three cell lines. Then, we detected whether MTA1 was recruited in the promoter region of the ERα gene and the influence of Z-LIG treatment using ChIP in MDA-MB-231 cells. Our result clearly showed that MTA1 was indeed recruited to the ERα promoter region. Importantly, Z-LIG decreased this recruitment (Figure 7B). To further confirm the role of MTA1 in the restoration of ERα expression by Z-LIG, we transfected MDA-MB-231 cells with pcMV-vector or pcMV-MTA1 before Z-LIG treatment. The result demonstrated that overexpression of MTA1 obviously reduced the ERα expression re-activated by Z-LIG in MDA-MB-231 cells (Figure 7C). Furthermore, our results also showed that overexpression of MTA1 reversed the inhibitory effect of cell viability of MDA-MB-231 cells treated by combinatorial Z-LIG and TAM (*p* < 0.01) (Figure 7D). In addition, colony formation assay also revealed that overexpression of MTA1 significantly reversed the suppression of combinatorial Z-LIG and TAM on the colony number of MDA-MB-231 cells (*p* < 0.01) (Figure 7E).

### Z-LIG decreased IFI16 and HDACs expression and recruitment to the ERα promoter

**Figure 8:**
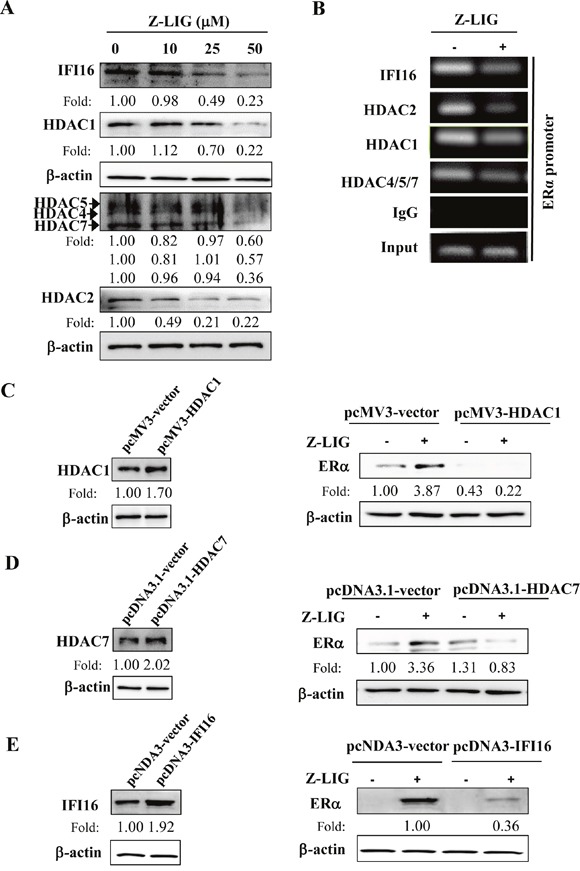
Z-LIG decreased IFI16 and HDACs expression and recruitment onto the ERα promoter in MDA-MB-231 **(A)** Expression of IFI16 and HDACs was detected by Western blotting in MDA-MB-231cells after treated with Z-LIG for 72 h. **(B)** ChIP analysis showing recruitment of IFI16 and HDACs onto ERpro315 of the ERα promoter. MDA-MB-231 cells were treated with Z-LIG (50 μM) for 72 h. DNA fragments that immunoprecipitated by normal IgG or or anti-IFI16, anti-HDAC1, anti-HDAC4/5/7, anti-HDAC2 antibodies antibodies were amplified by PCR using primers for ERpro315. **(C-E)** Expression of ERα was detected by Western blotting after MDA-MB-231 cells were transfected with vector and pcMV3-HDAC1, pcDNA3.1-HDAC7 or pcDNA-IFI16 for 24 h, andthen treated with Z-LIG (50 μM) for 72 h. β-actin was used as an internal control. The blots were a representative of three independent experiments.

IFI16 and HDACs are reported to be involved in the epigenetic regulation of ERα expression in ERα^−^ breast cancer cells [17, 35, 36]. Accordingly, we further examined the potential role of IFI16 and HDACs in our study. We first evaluated the expression change of IFI16 and HDACs in MDA-MB-231 cells treated by Z-LIG. As shown in Figure 8A, Z-LIG concentration-dependently decreased the expression of IFI16, which exhibits a relatively high basal level in MDA-MB-231 cells. Meanwhile, Z-LIG also reduced the expression of HDAC1, HDAC2 and HDAC4/5/7 in a similar way. Furthermore, we determined the level of recruitment of IFI16 and the selected HDACs in the promoter region of the ERα gene and the influence of Z-LIG treatment by ChIP. As a result, not only IFI16, but also HDAC1, HDAC2 and HDAC4/5/7 were recruited to the ERα promoter region. Moreover, the enrichment of IFI16 and the selected HDACs in the ERα promoter region was significantly reduced by Z-LIG (Figure 8B). These observations suggest both IFI16 and HDACs may be associated with the Z-LIG mediated restoration of ERα expression in ERα^−^ breast cancer cells. In previous studies, both class I HDACs and class II HDACs were found to be involved in the ERα regulation [17, 35, 36]. Thus, we further examined the potential role of class I HDACs (HDAC1 and HDAC2) and class II HDACs (HDAC4/5/7) in Z-LIG-mediated re-expression of ERα. As a representative, HDAC1 and HDAC7 were over-expressed in MDA-MB-231 cells before Z-LIG treatment. Figure 8C showed that overexpression of HDAC1 significant inhibited the ERα expression restored by Z-LIG. Similar result was also obtained when HDAC7 was over-expressed (Figure 8D). Moreover, our further study also showed that overexpression of IFI16 also remarkably reduced the ERα expression restored by Z-LIG (Figure 8E). These results indicate that both class I HDACs and class II HDACs, as well as IFI16 may be involved in Z-LIG-mediated re-expression of ERα in ERα^−^ breast cancer cells.

### Z-LIG destabilized MTA1/IFI16/HDACs containing NuRD complex in MDA-MB-231 cells

**Figure 9:**
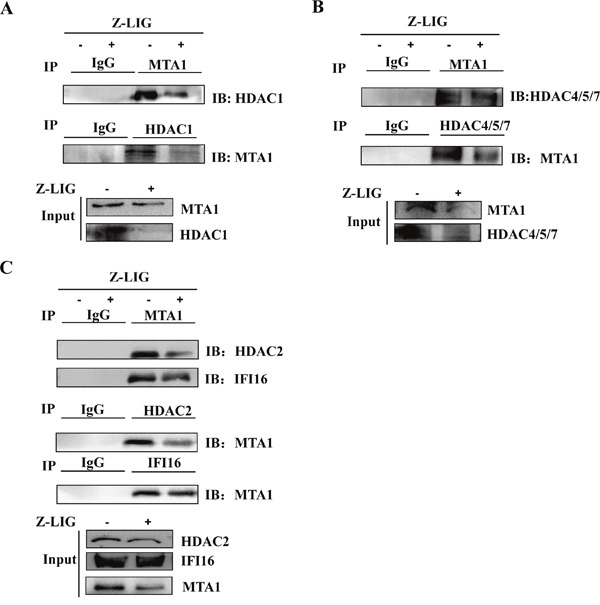
Z-LIG destabilized MTA1/IFI16/HDACs-containing NuRD complex in MDA-MB-231 cells **(A-C)** The whole cell lysates were immunoprecipitated (IP) with normal immunoglobulin G (IgG), anti-MTA1, anti-HDAC1, anti-HDAC4/5/7, anti-IFI16, anti-HDAC2 antibodies, and immunoprecipitates were fractionated and probed by Western blotting using anti-MTA1 and anti-HDAC1 **(A)** or anti-HDAC4/5/7 **(B)** or anti-IFI16, anti-HDAC2 **(C)** antibodies. The expression of MTA1, IFI16, HDAC1, HDAC2, and HDAC4/5/7 in whole cell lysates before immunoprecipitation was analyzed by Western blotting as input.

It has been shown that MTA1 in complex with IFI16 and HDACs contributes to epigenetic repression of ERα in ERα^−^ breast cancer cells [17]. We thereby examined MTA1/IFI16/HDACs interactions in MDA-MB-231 cells. Immunoprecipitation (IP) assay followed by Western blotting analysis of MTA1 or HDAC1 immunoprecipitate showed that there is a high physical association between MTA1 and HDAC1 (Figure 9A). Similar results were also observed between MTA1 and IFI16 or HDAC2 or HDAC4/5/7 (Figure 9B and 9C). These results suggest that a MTA1/IFI16/HDACs complex may be formed in MDA-MB-231 cells. Interestingly, these data showed that weaker association between MTA1 and IFI16 or HDACs in Z-LIG treated MDA-MB-231 cells compared to vehicle treated cells. Moreover, the level of MTA1, IFI16 and HDACs in whole lysate of MDA-MB-231 cells was also reduced after Z-LIG treatment, which is consistent with the results in Figure 8A.

## DISCUSSION

Hormonal therapies, such as TAM, is ineffective in patients with ERα^−^ breast cancer, which displays a more aggressive phenotype and a poorer prognosis [10]. Currently, the strategies to sensitizing ERα^−^ breast cancer to hormonal therapies are believed to be an effective and practical way [4, 10]. Emerging evidence strongly suggests epigenetic modification plays critical roles in the repression of ERα and the generation of hormone resistance in ERα^−^ breast cancer [4, 8, 17, 37]. Thus, novel approaches directed towards the key epigenetic factors contributing to the reactivation of ERα may provide an alternative way for sensitizing ERα^−^ breast cancer to hormonal therapies. In the present study, we aimed to investigate whether Z-LIG can reactivate ERα expression and restore TAM sensitivity. We first found that both VORAS and Z-LIG sensitized ERα^−^ breast cancer cells to TAM. Furthermore, Z-LIG combined with TAM induced the apoptotic cell death and induced S and G2/M phase cell cycle arrest. The growth inhibitory effect of TAM on ERα^+^ breast cancer cells is attributed to the competitive inhibition of the binding of estrogen to ERα, resulting in the repression of estrogen responsive genes [6, 7]. Thus, expression of ERα is necessary for response to TAM treatment. ERα^−^ breast cancer cells are found to be resistant to TAM due to the absence of ERα expression. Accordingly, we speculated that the re-sensitivity of ERα^−^ breast cancer cells by VORAS and Z-LIG to TAM in our study is closely related to the re-expression of ERα. As expected, both VORAS and Z-LIG reactivated the ERα expression in all the three ERα^−^ breast cancer cells used in our study, which provides an essential prerequisite for restoring the sensitivity to TAM. Furthermore, our results revealed that inhibition of ERα by ICI and si-ERα significantly prevent the growth inhibition of combinatorial Z-LIG and TAM on MDA-MB-231 cells, suggesting that ERα re-expression mediated by Z-LIG essentially contributes to sensitizing ERα^−^ breast cancer cells to TAM.

DNA methylation and chromatin remodeling are two epigenetic mechanisms that have been linked with the loss of ERα expression in ERα^−^ breast cancer [10, 37]. Previous studies have been shown that demethylation of the ERα promoter with 5-aza-dc or treatment with HDAC inhibitor TSA received promise in reactivating ERα expression in ERα^−^ breast cancer [11–13]. Additionally, several natural products such as (−)-epigallocatechin-3-gallate (EGCG) [36] and genistein [35], bioactive dietary combinations such as resveratrol and pterostilbene [38], green tea polyphenols (GTPs) and sulforaphane (SFN) [39] have also been reported to reactivate ERα expression independently or after combined with TSA. Notably, the histone active markers were commonly enriched by these natural products within the ERα promoter in ERα^−^ breast cancer cells, which leads to an open/more active chromatin structure [35, 36, 38]. In our study, we found that Ace-H3 (lys9/14) significantly increased after Z-LIG treatment in MDA-MB-231 cells. More importantly, Z-LIG remarkably promoted the enrichment of Ace-H3 (lys9/14) in the ERα promoter region, suggesting histone modification may contribute to Z-LIG mediated ERα re-expression in ERα^−^ breast cancer cells. MTA1, the founding member of the MTA family, plays key role in the NuRD complex [16, 40]. Interestingly, ERα was identified as the first direct target of MTA1 in 2001, establishing a direct connection between MTA1 and the NuRD complex in the transcriptional repression [41]. In general, MTA1 level is upregulated in human breast cancer with aggressive phenotypes [40, 42]. Moreover, overexpression of MTA1 was closely associated with TAM-resistance by blocking the transactivation activity of ERα [43]. Recently, a further study revealed that MTA1 transcriptionally represses the expression of ERα by recruiting class II HDACs along with the transcription factor IFI16 onto the ERα promoter [17]. Thus, we then determined the potential role of MTA1 in Z-LIG mediated re-expression of ERα and re-sensitivity to TAM in ERα^−^ breast cancer cells. It was interesting to see that Z-LIG not only reduced the MTA1 expression in the three ERα^−^ breast cancer cells, but also decreased the recruitment of MTA1 to the ERα promoter region in MDA-MB-231 cells. Moreover, overexpression of MTA1 significantly counteracted Z-LIG mediated re-expression of ERα. Further analysis revealed that overexpression of MTA1 reversed the inhibitory effect of combinatorial Z-LIG and TAM on cell viability and colony formation of MDA-MB-231 cells. IFI16, a DNA binding protein, is involved in multiple biological functions including induction of differentiation [44, 45], regulation of cell cycle [46] and activation of inflammasome response [47]. Earlier studies demonstrated that IFI16 was also identified as a transcriptional repressor [45]. Moreover, recent study demonstrated that IFI16 was involved in the MTA1-mediated repressor complex may contribute to the epigenetic repression of ERα expression in ERα^−^ breast cancer [17]. Our results showed that there is indeed a high basal level and an enrichment of IFI16 in the promoter region of the ERα gene in MDA-MB-231 cells, whereas Z-LIG reduced the IFI16 expression and its recruitment to the promoter region of the ERα gene. Accumulating evidence also showed that HDACs such as class I and class II HDACs are involved in the transcriptional repression of ERα via histone modification in ERα^−^ breast cancer [17, 35, 36]. We found that the expression of HDAC1, HDAC2 and HDAC4/5/7 was suppressed after Z-LIG treatment. Meanwhile, the recruitment of all these HDACs to the promoter region of the ERα gene was also reduced by Z-LIG. Moreover, our data clearly showed that both class I and class II HDACs may play critical roles in Z-LIG-mediated re-expression of ERα in ERα^−^ breast cancer cells. It has been shown that MTA1 together with IFI16 and class II HDACs was recruited and formed a complex, resulting in the epigenetic repression of ERα in ERα^−^ breast cancer cells [17]. Our observations indicate that there is obvious physical association for MTA1 with IFI16, or HDACs (HDAC1, HDAC2 and HDAC4/5/7), whereas Z-LIG remarkably reduced all these interactions. Additionally, it is worth noting that Z-LIG also decreased the expression of MTA1, IFI16 and HDACs (HDAC1, HDAC2 and HDAC4/5/7). These results suggest that Z-LIG resulted in decrease of the MTA1/IFI16/HDACs in the NuRD complex, likely leading to deregulation of their function.

**Figure 10:**
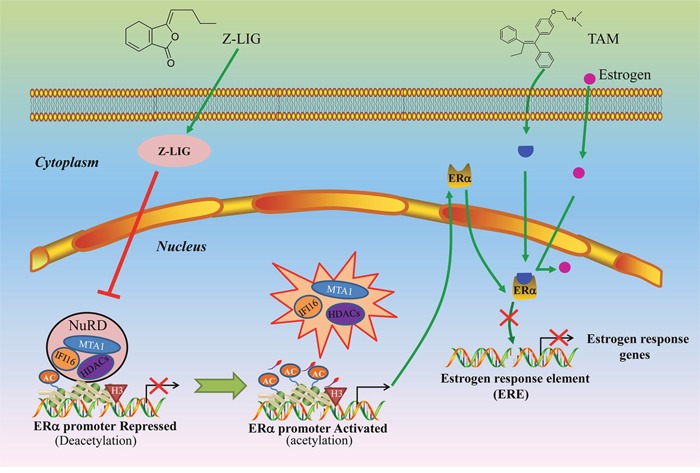
Proposed mechanism of re-expression of ERα and restoration of TAM sensitivity by Z-LIG in ERα^−^ breast cancer cells In the absence of Z-LIG, MTA1/IFI16/HDACs complexes are intact and repress transcriptional activation of ERα through deacetylation. Z-LIG leads to downregulation of MTA1, IFI16, HDACs (HDAC1, HDAC2, HDAC4/5/7) and subsequent destabilization of MTA1/HDAC1/HDACs interactions in NuRD complexes, which results in accumulation of ERα accessible for acetylation. Upon ERα re-expression, TAM as an estrogen antagonist competes with estrogen for binding to functional ER and then blocks the transcriptional activation of estrogen-sensitive genes, which will eventually lead to cell growth arrest.

Taken together, the results obtained in this study show that Z-LIG remarkably restored the inhibitory effect of TAM on ERα^−^ breast cancer cells. Combinatorial Z-LIG and TAM induced apoptosis and cell cycle arrest. Moreover, Z-LIG reactivated ERα expression and transcriptional activity. Finally, MTA1/IFI16/HDACs corepressor complex was identified as the key epigenetic mechanism regulated by Z-LIG for the reactivation of ERα and subsequent restoration of TAM sensitivity of ERα^−^ breast cancer cells (Figure 10). Thus, our study identified Z-LIG as a novel epigenetic modulator leading to reactivation of ERα expression and restoration of TAM sensitivity, which may have important clinical applications for ERα^−^ breast cancer chemoprevention and therapy.

## MATERIALS AND METHODS

### Materials

Z-LIG with purity more than 98 % was purchased from Chengdu Must Bio-Technology Co, Ltd (Chengdu, China) and stored in −80 °C before use. The antibodies against BRCA1, ERα, HDAC1, HDAC2, HDAC4/5/7, Acetyl-Histone3(lys9/14), IFI16, and MTA1 were purchased from Santa Cruz Biotechnology (CA, USA). Acetyl-p53, pro-PARP and cleaved PARP antibody were obtained from Cell Signaling Technology (Boston, MA, USA). The antibodies against Cyclin A, Cyclin E, CDK1, CDK2, p53, Histone 3 were obtained from Wanlei Biotechnology (Shenyang, China). The antibodies against caspase 3, cleaved caspase 3, p21 and p27 were purchased from Proteintech Group Inc (Wuhan, China). The antibodies against β-actin and rabbit IgG were obtained from Sigma-Aldrich (St. Louis, MO, USA). Other chemicals were obtained from Sigma-Aldrich, unless indicated otherwise.

### Cell culture

Human breast cancer cells MDA-MB-231, MDA-MB-453, HS578t were obtained from the American Type Cell Culture Collection (Manassas, VA). MDA-MB-231 cells were maintained in Dulbecco's modified Eagle's medium supplemented with 10% fetal bovine serum (FBS; Invitrogen, Carlsbad, CA) and 1% penicillin/streptomycin (Invitrogen). MDA-MB-453 and HS578t cells were cultured in RPMI1640 medium supplemented with 10% FBS and 1% penicillin/streptomycin. The three cell lines were cultured at 37 °C in a 5% CO_2_ and 95% air atmosphere.

### Measurement of cell viability

Survival rate of cells or cytotoxicity was measured by sulforhodamine B (SRB) assay (Sigma-Aldrich, St. Louis, MO, USA), which is based on the measurement of cellular protein content [48, 49]. Briefly, cells were stained with 0.4 % SRB for 30 min. The protein-bound dye was dissolved in 10 mM Tris base solution for OD determination at a wavelength of 490 nm using a multi-well spectrophotometer microplate reader (Biotek, Winooski, VT, USA). Cell viability was expressed as a percentage of that of the control (untreated) cells.

### Colony formation assay

Colony formation assay was performed as previously described [50]. MDA-MB-231, MDA-MB-453, HS578t Cells were seeded in a 6-well plate, respectively. Cells were pretreated with Z-LIG or vehicle for 2 days and then treated with Z-LIG and TAM alone or their combination for 14 days. The medium with corresponding compounds or vehicle was replaced per 3 days. At the end of treatment, cells were fixed in 100 % methanol and stained with 0.005% crystal violet. Finally, images were captured by a SONY camera (Tokyo, Japan) and the colonies were counted.

### Cell apoptosis analyzed by Flow cytometry

MDA-MB-231 cells were seeded and cultured overnight in 6-well plates. After treatment, cells were harvested, washed and re-suspended in the binding buffer containing annexin V and propidiumiodide (PI). After incubation at room temperature in the dark for 20 min, the stained cells were subjected to a BD LSRFortessa Cell Analyzer (BD Biosciences, San Jose, CA, USA) with fluorescence emission at 530 nm and 575 nm and excitation at 488 nm. Data were analyzed using Flow Jo 7.6.1 software (Tree Star, Inc., Ashland, OR, USA).

### Cell cycle analysis by flow cytometry

MDA-MB-231 cells were seeded and cultured overnight in 6-well plates. After treatment, cells were harvested, washed twice with ice cold PBS (pH 7.4) and fixed in 70 % ethanol for overnight at 4 °C. Then, cells were incubated with 250 μl of RNase A (100 μg/ml) for 30 min at 37 °C and finally stained with 500 μl of PI (50 μg/ml) for 1 h in the dark. Stained cells were analyzed with a BD LSRFortessa Cell Analyzer. Three independent experiments were performed. The relative percentages of cells in G1, S, or G2/M phase were calculated from FL-2 histograms using appropriate software (ModFit LT; BD, Topsham, ME, USA).

### Hoechst 33342 staining

MDA-MB-231 cells were seeded and cultured overnight in 12-well plate. After treatment, cells were washed with 1×PBS for 3 times. Then, Hoechst 33342 dissolving in 1×PBS was added into each well. The plate was kept at room temperature for 10 min and avoided from light. Finally, the plates were washed with 1×PBS again and images were captured under the fluorescence microscope (Nikon, Japan).

### Plasmids, siRNA duplexes, and transient transfection

PcMV3-HDAC1 was obtained from Sino Biological Inc. (Beijing, China). PcDNA3-FLAG-IFI16 (pcDNA3-IFI16) and pcDNA3.1-FLAG-HDAC7 (pcDNA3.1-HDAC7) were obtained from Addgen (MA, USA). PcMV-His-MTA1 (pcMV-MTA1) was purchased from GeneCopoeia (Guangzhou, China). Small interfering RNAs targeting ERα (si-ERα) were obtained from sigma (St. Louis, MO, USA). Cells were seeded into a 6-well plate at a density of 1.0×10^5^ cells/well and allowed to reach approximately 50 % confluence on the day of transfection. Then, cells were transfected with 50 nM siRNA or 2.5 mg DNA using transfection reagent Lipofectamine 2000 (Invitrogen, USA) according to the manufacturer's instructions. After a 6 h antibiotic-free medium incubation, the transfection medium was removed, and the cells were incubated in fresh medium for 24 h, followed by further drug treatments.

### Western blotting analysis

The total cellular proteins were extracted from cells with ice-cold RIPA buffer (Cell Signaling Technologies, USA) supplemented with 1% (v/v) protein inhibitor cocktail and 1 mM phenylmethylsulfonyl fluoride (PMSF). The cellular proteins (30 μg) were resolved by electrophoresis in 12 % SDS-polyacrylamide gel, and subsequently transferred to polyvinylidene difluoride (PVDF) membrane. Following 1 h incubation in a fresh TBS buffer containing 0.1% Tween-20 and 5% BSA, the blots were probed with specific primary antibodies. After incubation with the relevant secondary antibodies, the reactive bands were identified using an enhanced chemiluminescence (ECL) detection reagent (GE Healthcare, Sweden). The concentration of the loaded cellular proteins was normalized against the internal control β-actin, and then the value was expressed as each normalized data relative to control.

### Luciferase assay

The luciferase assay was performed as previously described [2]. The reporter construct 3×ERE TATA-Luc obtained from Addgen (MA, USA). It contains a firefly luciferase gene under the control of a consensus ERE site and premixed with constitutively expressing Renilla luciferase vector, which serves as an internal control for transfection efficiency. MDA-MB-231 cells were seeded in 6-well plates with a confluency of 70 %. Then, cells were transfected with the reporter plasmid by lipofectamine 2000 following the manufacturer's instructions. After 48 h of growth, cells were treated with drugs. Luciferase assays were performed using the Dual-Luciferase Reporter Assay System (Beyotime, China) according to the manufacturer's instructions. The firefly luciferase activity value was normalized to the Renilla activity value. Promoter activity was presented as a percentage of change compared with the vehicle-treated control.

### Chromatin immunoprecipitation (ChIP) assay

MDA-MB-231 cells were treated with 50 μM Z-LIG for 72 h. Approximately 1 × 10^6^ cells were cross-linked with a 1 % final concentration of formaldehyde (37 %, Sigma-Aldrich, St. Louis, MO, USA) for 10 min at 37 °C. ChIP assay was performed with the commercial kit according to the manufacturer's protocol (Beyotime, China). The epigenetic antibodies used in the ChIP assays were described above. ChIP-purified DNA was amplified by standard PCR using primers specific for the ERα promoter ranging from region +146 to +461 bp (ERpro315): sense, 5′-GCTGTGCTCTTTTTCCAGGT-3′ and anti-sense, 5′-GTCTGACCGTAGACCTGCGCGTTG-3′. PCR amplification was performed using the 2×PCR Master Mix (Promega, Madison, WI) and the reaction was initiated at 94 °C for 4 min followed by 30 cycles (94 °C, 30 s; 55 °C, 30 s; 72 °C, 1 min), and extended at 72 °C for 5 min. After amplification, PCR products were separated on 1.2 % agarose gels and visualized by Gel imaging system software (Tanon, Shanghai).

### Immunoprecipitation (IP) assay

MDA-MB-231 cells seeded in 100 mm plates were treated with Z-LIG or vehicle for 72 h. IP assay was carried out as the manufacturer's protocol (Beyotime, China). Cells were washed with 1×PBS and lysed in lysis buffer. The lysates were then centrifugated with 13000 g at 4 °C for 10 min. Fifty microliter of the samples was saved for normalization. One milliliter of lysates was immunoprecipitated with either IgG or antibody overnight at 4 °C. Immune complexes were pulled down using 40 μl of Protein A agarose plus beads and washed for 1-3 h. The immune complexes were then eluted with 30 μl of SDS sample loading buffer and subjected to Western blotting as described above.

### Trypan blue dye exclusion test

The growth inhibitory effect of Z-LIG and TAM alone or their combination on MDA-MB-231, MDA-MB-453 and HS578t cells were determined using trypan blue solution (sigma, St. Louis, MO, USA). Cells were treated separately for 24, 48, 72, 96 hours and then stained with trypan blue (0.4 %). The viable cells were counted using a hemocytometer.

### Statistical analysis

All data were presented as mean ± SD for three independent experiments. A ANOVA test was used to calculate the significant difference in the study. A *p*-value of less than 0.05 was considered to be statistically significant. All calculations were performed using the SPSS program, version 16.0.2 (SPSS Inc., Chicago, IL, USA).

## SUPPLEMENTARY MATERIALS FIGURES AND TABLES



## Abbreviations

ERα: Estrogen receptor alpha
TAM: Tamoxifen
Z-LIG: Z-ligustilide
IFI16: IFN-γ–inducible protein 16
HDACs: Histone deacetylases
MTA1: Metastasis-associated protein 1
ERE: Estrogen response elements
5-aza-dc: 5-aza-20-deoxycytidine
TSA: trichostatin A
NuRD: nucleosome remodeling and histone deacetylase
TCM: traditional Chinese medicine
VORAS: volatile oil of *Radix Angelica Sinensis*
Nrf2: nuclear factor-erythroid-2-related factor 2
SRB: Sulforhodamine B
CDK: Cyclin dependent dependent kinase
acetyl-p53: acetylation of p53
ICI: ICI 182780
Ace-H3: acetylation of histone H3
pcMV-MTA1: pcMV-His-MTA1
pcDNA3.1-HDAC7: pcDNA3.1-FLAG-HDAC7
pcDNA3-IFI16: pcDNA3-FLAG-IFI16
IP: immunoprecipitation
EGCG: (−)-epigallocatechin-3-gallate
SFN: sulforaphane
PI: propidiumiodide
PMSF: phenylmethylsulfonyl fluoride
PVDF: polyvinylidene difluoride
ECL: chemiluminescence
ChIP: Chromatin immunoprecipitation
