# Supplementary material for: Z-ligustilide restores tamoxifen sensitivity of ERα negative breast cancer cells by reversing MTA1/IFI16/HDACs complex mediated epigenetic repression of ERα
Source: Oncotarget. 2017 Mar 22;8(17):29328–45. doi: 10.18632/oncotarget.16440 (PMC5438733; doi:10.18632/oncotarget.16440)
Supplement: Supplementary file 1 [file oncotarget-08-29328-s001.pdf]

Z-ligustilide restores tamoxifen sensitivity of ERα negative breast cancer cells by reversing MTA1/IFI16/HDACs complex mediated epigenetic repression of ERα

SUPPLEMENTARY DATA

SUPPLEMENTARY METHODS

Extraction of VORAS from *Radix Angelicae Sinensis*

Radix Angelicae Sinensis was purchased from Sichuan Neautus Traditional Chinese Medicine Co., Ltd., which is a GMP certified pharmaceutical producer of herbal slices in China. The voucher specimens are deposited in our laboratory. Radix Angelicae Sinensis was beaten into coarse powder, and then over 20 mesh sieve, we took 58 g as a raw material, soak in water for 12 h, reflux extraction 12 h. Finally, we get 0.261 g of Angelica volatile oil. After the analysis of the high performance liquid chromatography, the purity ≥98 %.

SUPPLEMENTARY FIGURES AND TABLE

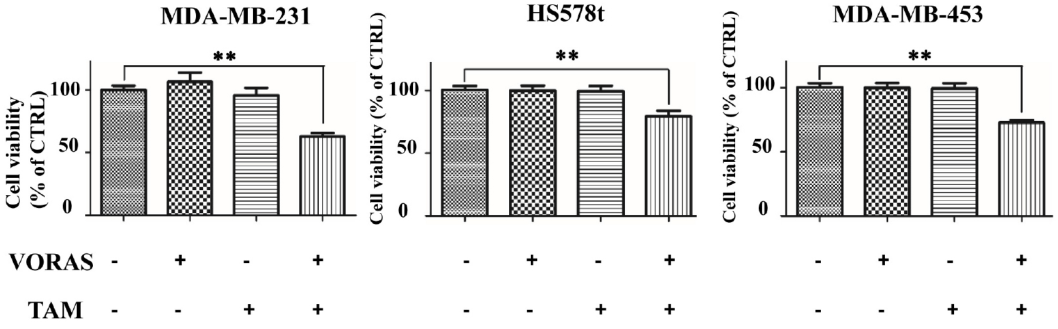

Supplementary Figure 1: Combined treatment of VORAS with TAM induced inhibitory effect on the cell viability of three ERα$^-$ breast cancer cell lines. MDA-MB-231, HS578t and MDA-MB-453 cells were pre-treated with VORAS (20 μ g/mL) for 12 h respectively. Then, VORAS (20 μg/ml) and TAM (5 μM) alone or combined for 72h. The cell viability were detected by SRB.

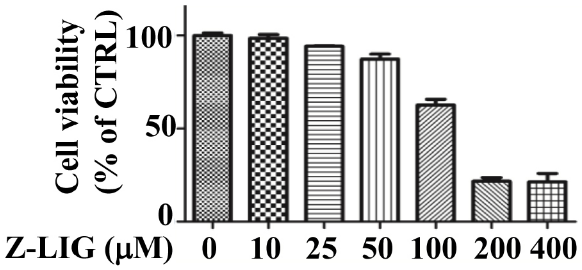

Supplementary Figure 2: Effect of Z-LIG on the cell viability of MDA-MB-231 cells. Cells were treated with Z-LIG as indicated for 72 h. Cell viability was determined with SRB assay.

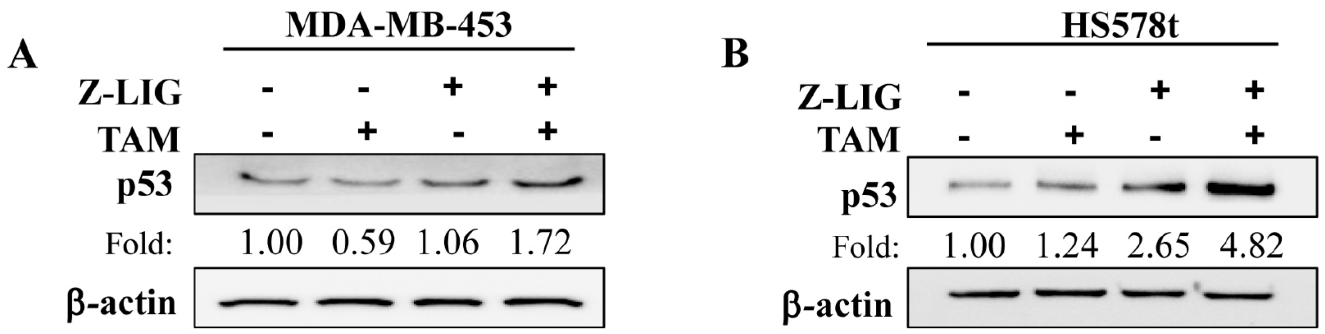

Supplementary Figure 3: Combinatorial Z-LIG and TAM increased the expression of p53. MDA-MB-453 and HS578t cells were treated with Z-LIG (50 μM) and TAM (5 μM) alone or their combination for 72 h. The expression of p53 was determined by Western blotting. β-actin was used as an internalcontrol. The blots were a representative of three independent experiments.

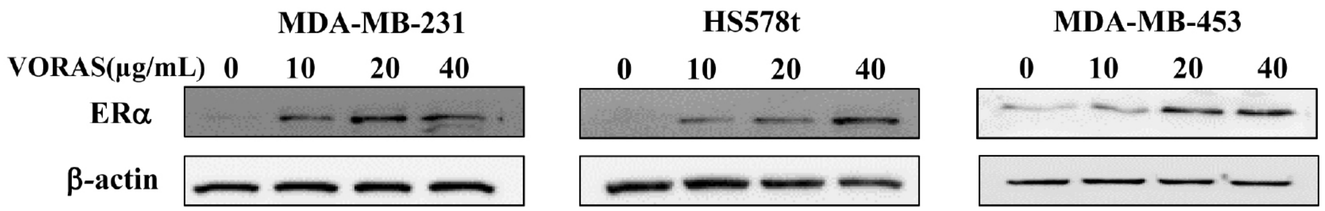

Supplementary Figure 4: VORAS induced ERα re-expression in three ERα⁻ breast cancer cell lines. MDA-MB-231, HS578t and MDA-MB-453 cells were treated with VORAS (10,20,40 μg/mL) for 72h. Then, ERα were detected by immunoblotting and the results were a representative of three independent experiments.

Supplementary Table 1: Cell cycle distribution (%)

Groups	G1	S	G2/M
CTRL	57.99±2.11	31.01±3.09	11.00±1.06
TAM	66.16±4.18	22.45±0.73	11.39±1.88
Z-LIG	30.80±2.72	50.51±1.76	19.41±1.66
Z-LIG+TAM	26.57±1.26	44.79±2.25	28.63±3.57

Z-LIG combined with TAM induces cell cycle arrest. MDA-MB-231 cells were treated with TAM and Z-LIG alone or combination as previous described, the cells were stained with PI solution, and 10000 events per experiment were analyzed using a flow cytometer. Values represent mean ± SD with three replicates. 0.01<*P < 0.005, **P < 0.001compared with control.
